# Pneumatosis cystoides intestinalis induced by sunitinib therapy in a patient with metastatic renal cell carcinoma: A case report

**DOI:** 10.1097/MD.0000000000039075

**Published:** 2024-07-26

**Authors:** Dong Jin Park, Donghyoun Lee, In Kyu Park

**Affiliations:** a Department of Surgery, Ulsan University Hospital, University of Ulsan College of Medicine, Ulsan, Republic of Korea; b Department of Surgery, Jeju National University Hospital, Jeju National University College of Medicine, Jeju, Republic of Korea

**Keywords:** clear-cell metastatic renal cell carcinoma, conservative treatment, pneumatosis cystoides intestinalis, sunitinib

## Abstract

**Introduction:**

Pneumatosis cystoides intestinalis (PCI) is a rare condition characterized by multiple gas-filled cysts in the gastrointestinal tract and is associated with numerous conditions. Benign PCI can occur secondary to certain medications, such as anticancer-targeted therapies. Here, we report a rare case of PCI that developed following sunitinib therapy for metastatic RCC and was successfully managed with conservative treatment without surgery.

**Patient concerns:**

A 57-year-old woman with a medical history of metastatic renal cell carcinoma (RCC) referred to the Department of General Surgery after completion of the 16th cycle of sunitinib because of abnormal findings on abdominopelvic computed tomography (CT), suggesting necrotizing enteritis with pneumoperitoneum involving the ileum. At the time of presentation to the Department of General Surgery, she was asymptomatic and had no abnormal findings on examination other than the imaging findings.

**Diagnosis:**

Sunitinib-induced PCI, metastatic RCC, liver cirrhosis, and diabetes mellitus.

**Interventions:**

She was admitted to the general ward for conservative treatment, and sunitinib was discontinued. Conservative treatments included nil per os, total parenteral nutrition, antibiotics, H2-blockers, and oxygen therapy.

**Outcomes:**

On the fifth day of hospitalization, the PCI showed moderate resolution on plain radiography, and she was discharged on the seventh day. Follow-up CT imaging 3 months later demonstrated complete resolution of PCI.

**Conclusion:**

This case emphasizes that the decision between conservative versus surgical treatment for PCI should be based not solely on radiological findings but rather on a comprehensive assessment, including the underlying condition, vital signs, physical examinations, and blood tests.

## 1. Introduction

Pneumatosis cystoides intestinalis (PCI; also known as peritoneal pneumatosis, pneumatosis cystoides intestinorum, abdominal gas cysts, cystic lymphopneumatosis, or intestinal gas cysts) is a rare condition characterized by multiple gas-filled cysts in the gastrointestinal tract, either subserosal, submucosal, or both^.[1]^ PCI is not a disease but a sign and is associated with numerous conditions.^[2]^ The etiology and pathogenesis of PCI have been investigated for decades, and multiple explanations have been proposed. Depending on the pattern of occurrence, they are classified into benign and fulminant forms, which can be further classified into primary and secondary.^[1–5]^ Benign PCI can occur secondary to certain medications, such as anticancer-targeted therapies.^[6–10]^ Sunitinib, a tyrosine kinase inhibitor, is a molecular-targeting agent used to treat gastrointestinal stromal tumors (GISTs) and metastatic renal cell cancer (RCC). Here, we report a rare case of PCI that developed following sunitinib therapy for metastatic RCC and was successfully managed with conservative treatment without surgery.

## 2. Case presentation

**Figure 1.**
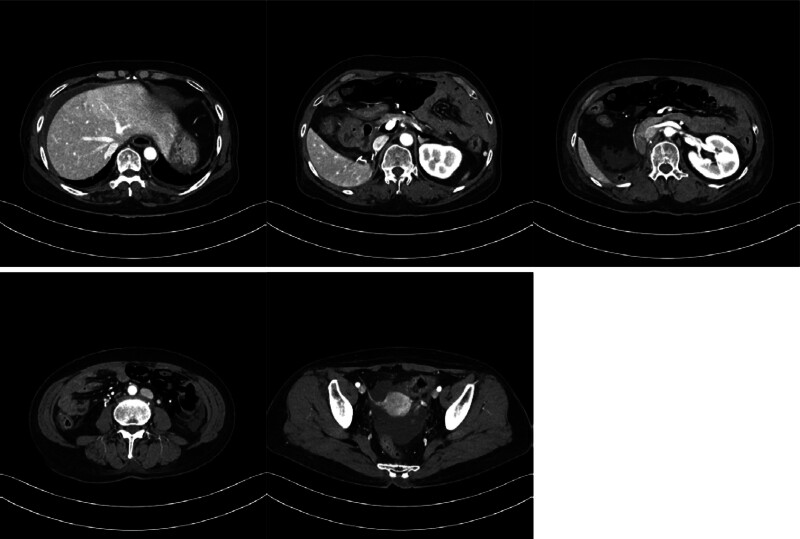
Abdominopelvic CT performed on the day of admission.

A 57-year-old female patient with a medical history of metastatic RCC, liver cirrhosis, and diabetes mellitus presented to the Department of General Surgery with abnormal findings on an abdominopelvic computed tomography (CT) scan conducted during routine surveillance of metastatic RCC. The patient’s medical history included an initial diagnosis of RCC at the age of 53 years when she underwent a workup for gross hematuria. Subsequently, she underwent radical nephrectomy by hand-assisted laparoscopic surgery at the Department of Urology. Pathological examination of the specimen revealed clear-cell RCC, WHO/ISUP grade 3, measuring 12.5 × 10.3 × 8.5 cm, staged as pT3aNx. Approximately 18 months postoperatively, a chest CT scan during regular follow-up revealed multiple lung metastases, prompting the initiation of targeted therapy with sunitinib (Sutene). The patient completed 15 cycles of sunitinib without any notable adverse effects. However, after the completion of the 16th cycle, an abdominopelvic CT scan revealed findings suggestive of necrotizing enteritis with pneumoperitoneum involving the ileum (Fig. 1). She was referred for surgical consultation from the Department of Urology. At the time of presentation to the Department of General Surgery, she was asymptomatic with normal vitality, and all blood tests were within normal limits (WBC 4930/μL, Hgb 11.4 g/dL, Platelet 53,000/μL). Physical examination revealed a soft and flat abdomen without localized tenderness, rebound tenderness or abdominal wall rigidity.

**Figure 2.**
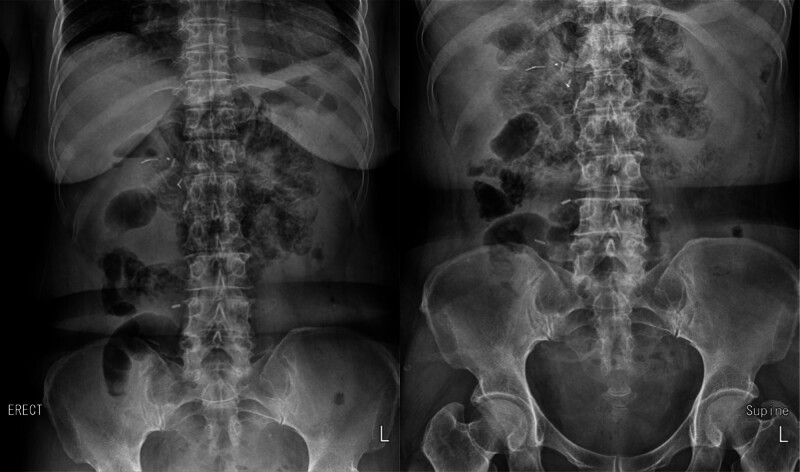
Plain X-ray of the abdomen (erect and supine) performed on the first day of hospitalization.

**Figure 3.**
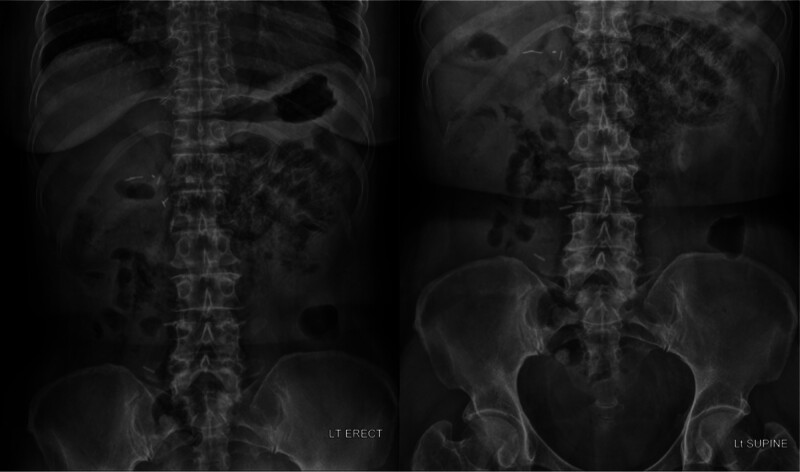
Plain X-ray of the abdomen (erect and supine) performed on the third day of hospitalization.

**Figure 4.**
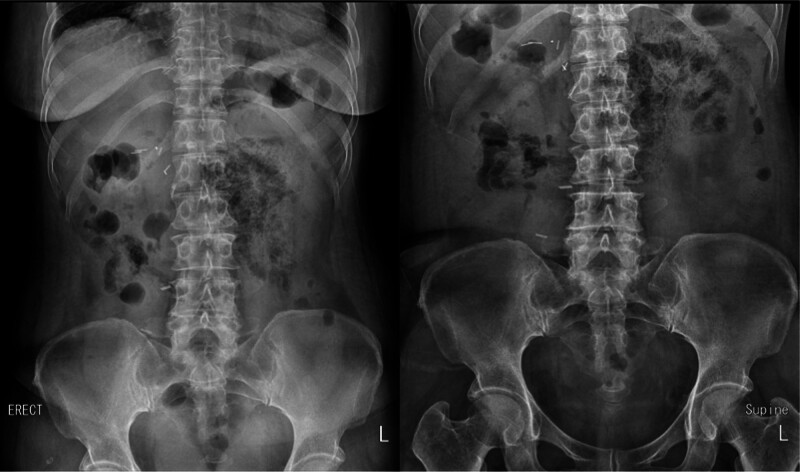
Plain X-ray of the abdomen (erect and supine) performed on the fifth day of hospitalization.

**Figure 5.**
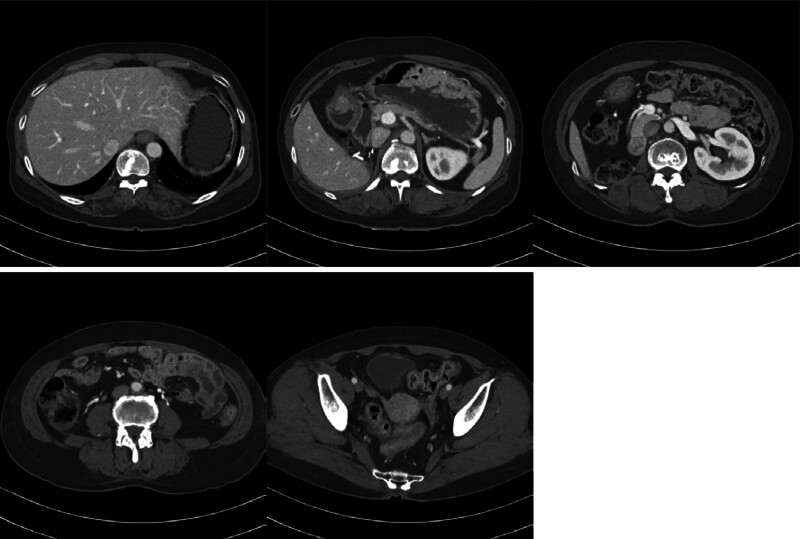
Abdominopelvic CT performed 3 months after discharge.

She was admitted to the Department of General Surgery for conservative treatment, and sunitinib was discontinued. Conservative treatment included nil per os (NPO), total parenteral nutrition, antibiotics, H_2_-blockers, and oxygen therapy. Blood tests and plain x-rays of the abdomen (erect and supine) were performed on the first (Fig. 2), third (Fig. 3), and fifth day of hospitalization (Fig. 4). Oral diabetes drugs were discontinued during the NPO period, and blood glucose levels were controlled with rapid-acting insulin. The patient remained asymptomatic throughout hospitalization, and all blood test results were within normal limits. PCI was observed on plain radiography of the abdomen until the third day of hospitalization, which moderately resolved on the fifth day. She was discharged on the seventh day of hospitalization. Approximately 3 months later, a follow-up CT scan showed complete resolution of the PCI without complications or sequelae (Fig. 5.). Following confirmation of the CT results, the patient was reintroduced to targeted therapy with axitinib (Inlyta) by the Department of Urology. To date, there have been no signs of worsening pulmonary metastases or PCI recurrence, and the patient remains under observation without adverse findings.

## 3. Discussion

PCI was first described by DuVernoi in 1730 following a cadaver dissection,^[11]^ and the term “PCI” was coined by Meyer in 1825.^[12]^ The etiology and pathogenesis of PCI remain unclear despite decades of investigation. Several theories have been proposed, of which mechanical, mucosal damage, bacterial, and pulmonary hypotheses seem to be the most promising.^[13]^ While the precise mechanism by which tyrosine kinase inhibitors (TKIs) induce PCI is not fully understood, it is believed to involve the inhibition of tyrosine kinases, which play crucial roles in maintaining the gastrointestinal epithelium’s integrity. One proposed mechanism is the TKI-driven disruption of the balance between apoptosis (cell death) and proliferation of intestinal epithelial cells,^[14]^ leading to the accumulation of gas within the intestinal wall and the formation of cysts. Furthermore, TKIs can affect the microvasculature of the gastrointestinal tract,^[15]^ leading to mucosal ischemia and subsequent gas accumulation in the submucosal or subserosal layers. Overall, the development of PCI in patients receiving TKIs likely involves a combination of factors, including the disruption of epithelial cell homeostasis, altered vascular perfusion, and mucosal injury, although further research is needed to elucidate the exact mechanisms fully.

Only a few cases of PCI caused by TKIs have been reported in the literature, with the majority recovering after conservative treatment.^[8,16,17]^ Only 2 cases have been reported wherein intra-abdominal findings were confirmed surgically. In 1 patient, after conservative treatment, thinning of the intestine was observed during the resection of a recurrent paracaval lymph node,^[17]^ while in the other, there was perforation of the jejunum.^[18]^ Since PCI is a sign rather than the disease itself and, in most cases, resolves with conservative management, such as the discontinuation of sunitinib, it can be inferred that treating the underlying condition leads to the resolution of PCI. Therefore, the decision to opt for conservative or surgical treatment should be based not solely on radiological findings but rather on a comprehensive assessment, including the underlying condition, vital signs, physical examinations, and blood tests. In the present case, PCI was incidentally discovered during a surveillance CT for RCC. As the patient was asymptomatic with stable vital signs and had no specific abnormal findings on physical examination and blood tests, conservative treatment was initiated after discontinuing the causative medication. Consequently, the PCI was significantly resolved by the fifth day of hospitalization and was completely resolved on a CT scan 3 months later. Furthermore, according to mechanical theory, gas dissects into the wall of the bowel from either the luminal surface through breaks in the mucosa or through the serosal surface by tracking along the mesenteric blood vessels.^[19]^ Once inside the bowel wall, the gas may spread along the mesentery to distant sites and can be associated with pneumoperitoneum or free air in the peritoneal cavity.^[20]^ In cases with intraperitoneal free air, as described above, if the patient is stable and there are no signs of peritonitis, conservative treatment should be prioritized over surgical intervention for the benign form of secondary PCI.

## 4. Conclusion

Here, we report a rare case of PCI in a patient with metastatic RCC that may be associated with sunitinib therapy. Conservative management led to the complete resolution of the condition without complications. This case highlights the importance of recognizing and managing the rare adverse effects of targeted therapy in cancer patients. Further research is warranted to elucidate the underlying mechanisms and optimal management strategies for this rare complication.

## Author contributions

**Writing – original draft:** Dong Jin Park, Donghyoun Lee, In Kyu Park.

**Writing – review & editing:** Dong Jin Park, Donghyoun Lee, In Kyu Park.

**Conceptualization:** In Kyu Park.

**Investigation:** In Kyu Park.

**Validation:** In Kyu Park.

**Visualization:** In Kyu Park.
